# Impact of size at birth and postnatal growth on metabolic and neurocognitive outcomes in prematurely born school-age children

**DOI:** 10.1038/s41598-021-86292-1

**Published:** 2021-03-25

**Authors:** Yoo Jinie Kim, Seung Han Shin, Eun Sun Lee, Young Hwa Jung, Young Ah Lee, Choong Ho Shin, Ee-Kyung Kim, Han-Suk Kim

**Affiliations:** 1 Department of Pediatrics, Seoul National University College of Medicine, Daehak-ro, Jongno-gu, Seoul, 101 Republic of Korea; 2 Department of Pediatrics, Seoul National University Bundang Hospital, Sungnam-Si, Republic of Korea

**Keywords:** Paediatric research, Metabolic syndrome

## Abstract

Prematurity, size at birth, and postnatal growth are important factors that determine cardiometabolic and neurodevelopmental outcomes later in life. In the present study, we aimed to investigate the associations between the size at birth and growth velocity after birth with cardiometabolic and neurodevelopmental outcomes in preterm infants. Fifty-six preterm infants born at < 32 weeks of gestation or having a birth weight of < 1500 g were enrolled and categorized into small for gestational age (SGA) and appropriate for gestational age (AGA) groups. Anthropometric and cardiometabolic parameters were assessed at school-age, and the Korean Wechsler Intelligence Scale for Children, fourth edition (K-WISC-IV) was used for assessing the intellectual abilities. The growth velocity was calculated by changes in the weight z-score at each time period. Multivariate analysis was conducted to investigate the associations of growth velocity at different periods with cardiometabolic and neurodevelopmental outcomes. Forty-two (75%) were classified as AGA and 25% as SGA. At school-age, despite the SGA children showing significantly lower body weight, lean mass index, and body mass index, there were no differences in the cardiometabolic parameters between SGA and AGA groups. After adjusting for gestational age, birth weight z-score, weight z-score change from birth to discharge and sex, change in weight z-score beyond 12 months were associated with a higher systolic blood pressure, waist circumference, and insulin resistance. Full-scale intelligent quotient (β = 0.314, *p* = 0.036) and perceptional reasoning index (β = 0.456, *p* = 0.003) of K-WISC-IV were positively correlated with postnatal weight gain in the neonatal intensive care unit. Although cardiometabolic outcomes were comparable in preterm SGA and AGA infants, the growth velocity at different time periods resulted in different cardiometabolic and neurocognitive outcomes. Thus, ensuring an optimal growth velocity at early neonatal period could promote good neurocognitive outcomes, while adequate growth after 1 year could prevent adverse cardiometabolic outcomes in preterm infants.

## Introduction

Metabolic syndrome is known as one of risk factors that develop cardiovascular disease and type 2 diabetes mellitus. Though no consensus has been made on the diagnostic criteria in the pediatric population, it presents as a combination of central obesity, hypertension, high triglyceride levels, and low high-density lipoprotein cholesterol levels, and glucose intolerance. After Barker^1^ proposed that fetal and postnatal environment and growth could be contributing factors of metabolic syndrome, research on small for gestational age (SGA) and preterm infants has increased.

Several studies have reported SGA as a risk factor for adverse metabolic consequences in later life of term infants^2–5^. According to a Polish study^2^, children at pre-pubertal age who were born as SGA at term showed a high prevalence of particular components of metabolic syndrome. Numerous studies have also identified the effects of preterm birth on metabolic outcomes^6–11^. A recent systematic review and meta-analysis reported that preterm birth was associated with higher fat mass, blood glucose, insulin resistance, and total cholesterol level in adults^7^.

The role of SGA in metabolic outcomes in later life among preterm infants has been demonstrated in several studies. Huang et al.^12^ reported the risk of metabolic syndrome in preterm and/or SGA children. Lower insulin sensitivity at pre-pubertal age^13^ and elevated blood pressure after 31 years of follow-up^14^ were observed in preterm infants born as SGA, as compared to appropriate for gestational age (AGA) group. In contrast, other studies demonstrated no differences in the cardiometabolic outcomes between the two groups of preterm infants^15,16^; like Darendelier et al. reported that there were no differences in insulin resistance and body composition during the pre-pubertal period between preterm SGA and AGA^17^.

Accelerated growth is another important risk factor for metabolic syndrome in the later part of life in term SGA infants^18^. On the contrary, slow weight gain was found to result in adverse neurodevelopmental outcomes in term SGA infants^19^. Likewise, the influence of postnatal growth on an increased risk of metabolic syndrome in preterm infants has been demonstrated as well^20,21^. In addition, unfavorable neurodevelopmental outcomes following poor growth have also been reported in this population^22,23^. However, the optimal intrauterine and extrauterine growth patterns related to cardiometabolic and neurocognitive outcomes have not been established in preterm infants so far.

In this study, the prevalence of adverse cardiometabolic findings at school-age was evaluated among very preterm infants born as AGA and SGA. Moreover, to investigate the role of growth velocity at different periods on health outcomes of preterm infants, the associations between postnatal growth velocity at different periods with the health status at school-age were analyzed in the study population, including cardiometabolic and neurodevelopmental outcomes.

## Methods

### Study design

This was a retrospective cohort study. Preterm infants born at < 32 weeks of gestation or having a birth weight of < 1,500 g and who were admitted to the neonatal intensive care unit (NICU) at Seoul National University Children’s Hospital between 2008 and 2009 were enrolled in the cohort at a chronological age of 6–8 years. Among 266 infants who were born below 32 weeks’ gestation or 1,500 g at birth, families of 136 infants were contacted, and 60 of them consented to participate in the cohort. Of the 60 infants in the cohort, four with grade IV intraventricular hemorrhage (IVH), according to the classification by Papile et al.^24^ or periventricular leukomalacia (PVL) were excluded. SGA was defined as a birth weight of < 10th percentile for age by the Fenton growth chart^25^. The participants were assigned to two groups, SGA and AGA, based on the birth weight. This study was approved by the Institutional Review Board of Seoul National University Hospital. Written informed consent was obtained from the parents of all participants. All methods were performed in accordance with the guideline of the Human Research Protection Program.

### Data collection and laboratory analyses

Data on perinatal factors, including birth weight, gestational age (GA), delivery mode, sex, and multiple births were reviewed. Data on neonatal outcomes, such as treated patent ductus arteriosus (PDA), respiratory distress syndrome (RDS), moderate to severe bronchopulmonary dysplasia (BPD), and sepsis were collected. Information on the duration of breast milk feeding and additional calorie intake other than fortifier was also reviewed. Body weight at discharge, corrected age (CA) of 4 months, chronological age at 12 and 24 months, and school-age were measured. The z-scores of body weight were calculated according to the World Health Organization growth charts.

At school-age, anthropometric parameters, including weight, waist circumference (WC), and height were assessed. The fat mass was measured using the InBody Test (BIA; InBody 770, Biospace Co., Seoul, Korea). The body mass index (BMI) was calculated by dividing weight in kilograms by height in meters squared, and the fat mass index (FMI) was obtained by dividing the fat mass in kilograms by height in square meters. Lean mass index (LMI) was calculated by dividing the lean mass by the square of height. The blood pressure (BP) (systolic and diastolic) was also measured with an automated device at the left arm using an appropriate-sized cuff. The BP was measured twice, and the average value of the two readings was recorded. The BP was interpreted using the normative BP table proposed by Lee et al.^26^. The Korean national growth charts for children and adolescents released in 2007 were used to calculate the percentile of WC. The parents filled questionnaires related to the recent daily calorie intake and physical activity.

The blood sample of each participant was obtained by venipuncture after 8 h of fasting. The homeostasis model assessment of insulin resistance (HOMA-IR) representing the insulin resistance was evaluated after estimating fasting plasma insulin and glucose levels. Then, the values were applied to the following equation: HOMA-IR = fasting plasma insulin (mU/mL) × fasting plasma glucose (mg/dL) / 405. The lipid profiles including total cholesterol, triglyceride, high-density lipoprotein cholesterol (HDL-C), leptin, and adiponectin were also estimated. The intellectual abilities at 6 years of age, including verbal comprehension, perceptual reasoning, working memory, and processing speed, were tested using the Korean Wechsler Intelligence Scale for Children, fourth edition (K-WISC-IV), administered by a trained assessor.

### Statistical analyses

The data are presented as median (interquartile ranges) or count (percentage). The Wilcoxon rank-sum test was conducted to compare the continuous variables and Fisher’s exact test was used for the categorical variables. Multivariate linear regression analysis was conducted to investigate the association between the changes in the z-score at each epoch with the cardiometabolic factors at school-age. Regression coefficients beta (β) with *p* values were calculated after adjusting for GA, birth weight z-score, weight z-score change from birth to discharge, and sex. Multivariate linear regression analysis was also used to determine the effect of weight gain at different epochs on each index scale of K-WISC-IV after adjusting for factors that could influence neurocognitive outcomes, such as GA, birth weight z-score, weight z-score change from birth to discharge, sex, and IVH^27–30^. A *p* value of < 0.05 was considered statistically significant. STATA 12.0 for Windows (Stata Corp., College Station, TX, USA) was used to analyze all data.

### Consent

Written informed consent was obtained from the participants of the study.

## Results

### Subject characteristics

Among the 56 school-aged children born prematurely, 14 were born as SGA. The gestational age was higher in the SGA group than that in the AGA group (28 vs. 31.6 weeks, *p* < 0.001), and RDS was more prevalent in the AGA group (42.9% vs. 7.1%, *p* = 0.021) (Table 1). Otherwise, there were no differences in the perinatal characteristics between two groups. Although the postmenstrual age at discharge was comparable, weight at discharge was higher in the AGA group (2360 vs. 2095 g, *p* < 0.001).

**Table 1:** Perinatal and neonatal characteristics of the study population.

	AGA (n = 42)	SGA (n = 14)	*p* value
GA (week)	28 (26–29.6)	31.6 (30–32.9)	< 0.001
Birth weight (g)	960 (860–1250)	865 (720–1120)	0.116
Apgar score at 1 min	4.5 (2–6)	3.5 (2–5)	0.467
Apgar score at 5 min	7 (6–7)	7 (4–7)	0.576
Female	20 (47.6)	6 (42.9)	1.000
GDM	5 (11.9)	2 (14.3)	1.000
Preeclampsia	6 (14.3)	4 (28.6)	0.247
Cesarean section	27 (64.3)	12 (85.7)	0.186
Multiple birth	24 (57.1)	7 (50)	0.759
RDS	18 (42.9)	1 (7.1)	0.021
Moderate to severe BPD	11 (26.2)	2 (14.3)	0.480
Sepsis	5 (12.2)	2 (14.3)	1.000
NEC	1 (2.4)	1 (7.1)	0.441
IVH	24 (57.1)	7 (50)	0.759
PMA at discharge (week)	37.5 (35.6–39.9)	38.5 (37.4–42)	0.057
Weight at discharge (g)	2360 (2080–2760)	2095 (1880–2440)	< 0.001

Values are expressed as N (%) or Median (interquartile range).

Abbreviations: GDM, gestational diabetes mellitus; RDS, respiratory distress syndrome; BPD, bronchopulmonary dysplasia; NEC, necrotizing enterocolitis; IVH, intraventricular hemorrhage.

### Anthropometric parameters, blood pressure, laboratory findings, including lipid profile, and insulin resistance at school-age

At school-age, weight, BMI, lean body mass, and LMI were lower in the SGA group (Table 2). However, no significant differences in BP, lipid profiles, fasting glucose, insulin level, and HOMA-IR were found between two groups. Based on the questionnaires, no significant differences in calorie intake and activity were identified between two groups (Supplementary Table S1).

**Table 2:** Body measurements, blood pressures and laboratory findings at school-age.

	AGA (n = 42)	SGA (n = 14)	*p *value
Age (year)	7.2 (6.8–7.6)	7.1 (6.9–7.5)	0.784
Weight (kg)	21.5 (20.4–25)	19.8 (16.9–20.8)	0.015
BMI (kg/m^2^)	15 (14.1–16)	13.9 (12.1–14.9)	0.009
Waist circumference (cm)	53.95 (50.9–57)	52 (46.2–55)	0.191
Fat mass (kg)	3.2 (2.5–4.6)	2.6 (1.4–4)	0.104
Fat mass index (kg/m^2^)	2.2 (1.7–3)	1.8 (0.9–2.7)	0.150
Lean mass (kg)	18.8 (17.3–21.2)	16.8 (16.3–18.5)	0.005
Lean mass index (kg/m^2^)	12.8 (12.4–13.5)	12 (11.3–12.7)	0.006
Systolic BP (mmHg)	106 (99.5–109)	109.8 (104.5–115)	0.374
Diastolic BP (mmHg)	62 (55.5–65)	69.3 (66.5–72)	0.438
Cholesterol (mg/dL)	177 (163–194)	174.5 (152–194)	0.443
Triglyceride (mg/dL)	60.5 (45–75)	58 (53–67)	0.865
HDL-C (mg/dL)	70 (61–79)	68.5 (61–78)	0.850
Fasting glucose (mg/dL)	96 (93–100)	94 (91–100)	0.655
Insulin (uIU/mL)	4.4 (3.4–5.9)	3.6 (2.9–5.3)	0.325
HOMA-IR	1.04 (0.81–1.5)	0.86 (0.7–1.25)	0.482
Leptin (ng/mL)	6.2 (4.8–8.4)	6.2 (5.4–10.1)	0.777
Adiponectin (μg/mL)	9.7 (7.7–11.5)	8.2 (6.3–10)	0.108

Values are expressed as N (%) or Median (interquartile range).

Abbreviations: BMI, body mass index; HOMA-IR, homeostatic model assessment-insulin resistance; HDL-C, high-density lipoprotein cholesterol.

### Growth patterns from birth to school-age

The weight z-scores were lower in the SGA group from birth to school-age (Table 3). Although changes in the weight z-scores from 4 months CA to 12 months were significantly higher in the SGA group (-0.22 vs. 0.36, *p* = 0.028), there were no differences in the changes of z-scores during the other epochs. In both groups, the catch-up of weight z-score was mostly achieved during the period from discharge to 4 months CA.

**Table 3:** Growth patterns of the study population.

	AGA (n = 42)	SGA (n = 14)	*p* value
Weight z-score at birth	0.05 (− 0.51–0.32)	− 1.98 (− 2.6–1.63)	< 0.001
Weight z-score at discharge	− 1.33 (− 1.93–0.76)	− 3.74 (− 4.34–2.77)	< 0.001
Weight z-score at CA 4 months	0.31 (− 0.53–1.12)	− 1.6 (− 2.53–0.59)	< 0.001
Weight z-score at 12 months	0.09 (− 0.54–0.67)	− 0.98 (− 2.17–0.56)	< 0.001
Weight z-score at 24 months	− 0.18 (− 0.71–0.68)	− 1.37 (− 2.15–0.5)	0.001
Weight z-score at school− age	− 0.35 (− 1.1–0.37)	− 1.4 (− 2.4–0.8)	0.005
△ z-score weight (birth ~ discharge)	− 1.16 (− 1.76–0.89)	− 1.43 (− 1.89–0.97)	0.432
△ z-score weight (discharge ~ CA 4 months)	1.54 (1.13–2.2)	1.51 (0.78–2.47)	0.925
△ z-score weight (CA 4 months ~ 12 months)	− 0.22 (− 0.61–0.3)	0.36 (0.11–0.63)	0.028
△ z-score weight (12 months ~ 24 months)	− 0.2 (− 0.53–0.24)	0.02 (− 0.29–0.48)	0.329
△ z-score weight (24 months ~ school− age)	− 0.35 (− 0.75–0.2)	− 0.32 (− 1.25–1.43)	0.886

### Association of growth with cardiometabolic and neurocognitive outcomes at school-age

In the multivariate linear regression analysis after adjustment for GA, birth weight z-score, weight z-score change from birth to discharge, and sex, cardiometabolic outcomes were not associated with z-score changes before 12 months after birth (Table 4). Only the z-score of birth weight showed a positive correlation with diastolic BP at school-age. Waist circumference, fasting insulin, and HOMA-IR were positively associated with z-score changes during 12 − 24 months and 24 − 72 months. The HDL-C level was negatively correlated with the z-score changes from 12 − 24 months, and positive correlations were also observed between z-score changes from 24 months to school-age and the systolic BP, FMI, and leptin levels.

**Table 4:** Change in weight z-score in different epochs on outcome variables at school-age.

	Birth z-score		Birth to discharge		Discharge to 4 mo CA		4 mo CA to 12 mo		12 to 24 mo		24 mo to school-age	
	Beta	*p *value	Beta	*p* value	Beta	*p* value	Beta	*p* value	Beta	*p* value	Beta	*p* value
Systolic BP (mmHg)	− 0.001	0.997	0.152	0.298	− 0.052	0.729	− 0.024	0.871	− 0.009	0.958	**0.476**	**0.001**
Diastolic BP (mmHg)	**0.371**	**0.034**	0.259	0.064	− 0.044	0.755	0.120	0.396	− 0.098	0.535	0.248	0.075
Waist circumference (cm)^§^	0.260	0.147	0.212	0.146	0.155	0.292	0.176	0.236	**0.451**	**0.005**	**0.590**	** < 0.001**
Fat mass index (kg/m^2^)^§^	0.330	0.058	0.229	0.101	0.130	0.357	0.226	0.107	0.212	0.181	**0.600**	** < 0.001**
Fasting glucose (mg/dL)	− 0.106	0.548	− 0.189	0.186	0.184	0.202	0.007	0.962	0.135	0.432	0.249	0.095
Fasting insulin (uIU/mL)^§^	0.035	0.848	− 0.089	0.553	− 0.131	0.391	0.220	0.151	**0.433**	**0.010**	**0.534**	** < 0.001**
HOMA-IR^§^	− 0.058	0.751	− 0.108	0.472	− 0.106	0.491	0.221	0.152	**0.370**	**0.036**	**0.534**	** < 0.001**
Cholesterol (mg/dL)^§^	− 0.069	0.688	− 0.121	0.394	− 0.065	0.656	0.103	0.481	− 0.119	0.470	− 0.155	0.289
HDL-C (mg/dL)	− 0.088	0.618	− 0.138	0.343	− 0.084	0.570	− 0.040	0.787	− **0.484**	**0.003**	− 0.005	0.975
Leptin (ng/mL)^§^	− 0.071	0.689	0.138	0.348	− 0.060	0.689	0.247	0.098	0.205	0.184	**0.651**	** < 0.001**

Bold values indicates statistically significant results.

^§^Logarithmic transformation was performed as needed to improve normality. Data are presented as the regression coefficient beta from multivariate linear regression analysis. Adjusted for gestational age, birth weight z-score, weight z-score change from birth to discharge, and sex.

Abbreviations: HOMA-IR, homeostatic model assessment-insulin resistance; HDL-C, high-density lipoprotein cholesterol; mo, month; CA, corrected age.

The z-score changes in weight from birth to discharge were positively related with the full-scale intelligence quotient (FSIQ) (β = 0.314, *p* = 0.036) and perceptual reasoning index (β = 0.456, *p* = 0.003) at school-age (Table 5). However, the z-score changes after discharge at any periods, as well as birth weight z-score, did not show any correlation with FSIQ, as well as each index of K-WISC-IV.

**Table 5:** Change in weight z-score in relation to full scale and each index of K-WISC-IV.

	Birth z-score		Birth to discharge		Discharge to 4 mo CA		4 mo CA to 12 mo		12 to 24 mo		24 mo to school-age	
	Beta	*p* value	Beta	*p* value	Beta	*p* value	Beta	*p* value	Beta	*p* value	Beta	*p* value
Full scale IQ	0.030	0.395	**0.314**	**0.036**	− 0.031	0.826	0.091	0.523	− 0.229	0.144	0.006	0.966
Verbal comprehension	0.195	0.246	0.027	0.868	0.165	0.287	− 0.116	0.457	0.012	0.945	− 0.021	0.897
Perceptional reasoning	0.315	0.190	**0.456**	**0.003**	0.031	0.825	− 0.010	0.944	− 0.254	0.099	0.024	0.871
Working memory	0.128	0.286	0.089	0.575	− 0.131	0.392	0.263	0.084	− 0.126	0.464	− 0.096	0.550
Processing speed	0.001	0.564	0.211	0.110	− 0.210	0.089	0.176	0.162	− 0.265	0.061	0.172	0.196

Bold values indicates statistically significant results.

Abbreviations: K-WISC-IV, Korean Wechsler Intelligence Scale for Children, fourth edition; IQ, intelligent quotient; mo, month; CA, corrected age.

Data are presented as the regression coefficient beta from multivariate linear regression analysis.

Adjusted for gestational age, birth weight z-score, weight z-score change from birth to discharge, sex, and intraventricular hemorrhage.

## Discussion

In the present study, the effect of size at birth and postnatal growth at different epochs on the health status at school-age, including metabolic and neurodevelopmental outcomes, were assessed in preterm infants. Among the study population, the AGA group included more premature infants and had a higher prevalence of RDS compared to the SGA group. While weight, LMI, and BMI at school-age were lower in the SGA group, there were no differences in the cardiometabolic parameters, including BP and insulin resistance, between preterm infants born as SGA and AGA. Multivariate analysis showed that cardiometabolic outcomes were associated with weight gain after 12 or 24 months, while neurocognitive outcomes were associated with weight gain before discharge from the NICU.

Term SGA is considered as an important risk factor for developing metabolic syndrome in the later part of life^4,31^. Increased cardiovascular risk, shown as peripheral conduit arterial stiffness and higher mean systolic BP, was observed in children born preterm and SGA^32^. However, the effects of SGA at birth on later health outcomes in preterm infants have not been clearly demonstrated so far. In a cohort study performed in New Zealand, the prevalence of metabolic syndrome was comparable between very-low birth weight infants with or without SGA^15^. There were no differences in the systolic and diastolic BP of adults regardless of their birth weight among very-low birth weight infants in the aforementioned study^33^.

Such controversies on cardiometabolic outcomes in preterm SGA might be contributed by the growth velocity or catch-up growth of preterm SGA infants in each study, as rapid postnatal growth is known as a key component in the development of metabolic syndrome in term SGA infants^18^. Studies reporting SGA as a risk factor for metabolic syndrome among preterm infants also showed that preterm SGA achieved catch-up growth, reporting comparable weight or BMI with preterm AGA infants at evaluation^13,14^. However in the present study, although growth velocity was greater from 4 to 12 months of age in the SGA group, weight z-scores were consistently lower until school-age, accompanied by lower LMI and lower BMI at evaluation. In a term SGA study, risk of metabolic syndrome was reportedly lower in SGA infants without excessive catch-up growth during infancy, compared with those with catch-up growth^19^.

To clarify the role of growth velocity in the development of cardiometabolic abnormalities in the study population, multivariate analysis was conducted adjusting the birth weight z-score. The result showed that the growth velocity beyond 12 or 24 months of age was correlated with several cardiometabolic outcomes in our study. The critical period of growth for the later health outcomes in preterm infants has not yet been established. Several studies have reported the association of early postnatal growth patterns, especially until 3 months of CA, and unfavorable health outcomes in the later part of life. A Dutch national prospective follow-up study reported that early postnatal growth from birth to CA of 3 months in very preterm infants was associated with higher BMI, fat mass, and percentage of body fat at age 19 years^34^. A rapid catch-up during the first 3 months after term age was positively associated with higher fat percentage, waist circumference, and serum triglyceride level at 21 years of age^35^. As compared to these studies, the current study showed that relatively later growth after 12 months of life is related to abnormal findings of the cardiometabolic components at school-age. This is compatible with the report of Embleton et al.^36^, who reported the association of growth beyond 1 year of age and adverse metabolic outcomes in children who were born prematurely. Interestingly, the associations of earlier growth (< 3 months of CA) and later outcomes were found in a young adult study, while the effect of later growth (> 1 year of age) was found at a relatively young age, including the present study.

In terms of neurodevelopmental outcomes, changes in weight z-score during NICU stay had a positive correlation with full scales of K-WISC-IV, especially with the perceptional reasoning index in our study. On the other hand, there was no association between neurocognitive outcomes and z-score of birth weight or growth after discharge. Early postnatal growth of preterm infants is important for favorable neurodevelopmental outcomes. The association of in-hospital weight gain of preterm infants and improvement of mental processing composite score of the Kaufmann Assessment Battery at 5 years of age was demonstrated by Franz et al.^37^. According to Ehrenkranz et al., better weight growth during NICU stay reduced the incidence of cerebral palsy and poor neurocognitive outcomes at 18 − 22 months’ of CA^38^.

The trade-off phenomenon by postnatal growth in preterm infants has been well demonstrated in two multi-center longitudinal studies from the Infant Health and Development Project in the US. In the first study, rapid weight gain from term to age 4 months was associated with better cognitive outcomes at school-age and higher BP^21^. Another study reported that linear growth from term to 4 months was related to better cognitive outcomes at 8 years and 18 years of age, while the risk of becoming overweight or obese was increased at 8 years of age^39^. However, these previous studies did not consider the role of postnatal growth in the NICU.

In the present study, the growth during NICU stay was calculated and adjusted because postnatal growth before term equivalent age plays an important role in cardiometabolic outcomes^40–42^ and neurodevelopmental outcomes^43,44^. Deprivation followed by a relatively enriched environment is a pivotal hypothesis of the developmental origin of adult disease^1^. The postnatal period during NICU stay in preterm infants could be understood as a deprived environment because many very preterm infants experience extrauterine growth restriction during this period^45,46^, and it might be considered in the analysis as an effect of growth after the neonatal period on later health outcomes.

The results of the present study might provide some clues to set an optimal growth target and accordant nutritional interventions for preterm infants because many term SGA studies reported postnatal growth as a double-edged sword between health in later life and neurodevelopmental outcomes. However, if growth at different periods affects different aspects of later health outcomes, nutritional interventions could be focused at a time when these could lead to favorable outcomes^47–49^.

There are limitations to generalizing the results of the present study. This is a single-center study with a small number of children. Only 22.5% of the original birth population constituted the study cohort, and 14 out of 56 preterm infants were born as SGA. Moreover, control group was not included in the current study. Therefore, the statistical power of the present study was low. However, the growth pattern of preterm and term infants should be compared with caution because most preterm infants experience postnatal growth restriction immediately after birth and growth standards regarding later health outcomes have not been established. In this respect, the purpose of the current study was confined to the evaluation of the association between growth velocity and health outcomes among preterm population.

In the present study, intrauterine growth restriction (IUGR) was not assessed among the study population. Although IUGR is often used interchangeably with SGA, their definitions and etiologies are different. In addition, an IUGR fetus may be born as either SGA or AGA and vice versa. IUGR is defined as a lower-than-normal fetal growth rate, considering the growth potential by fetal race and sex, whilst SGA is determined by size at birth, such as weight and height according to the sex^50,51^. SGA could originate from factors other than obstetric and fetal problems, such as genetic causes (small parents) and maternal weight gain during pregnancy^52^. However, SGA could be used as a proxy of IUGR, and many studies also reported adverse cardiometabolic outcomes in the SGA population^2,4,5,19,31,32^.

Although growth velocity during NICU stay was associated with favorable later neurocognitive outcomes, optimal standards for growth could not be driven from this study only. Prospective studies with larger populations are required to establish optimal growth standards for adulthood health outcomes, including both metabolic and neurodevelopmental outcomes. Moreover, longitudinal follow-up study evaluating the components of the metabolic syndrome and actual diagnosis of the disease at older age is needed.

## Conclusion

In the present study, among preterm infants, more rapid weight gain during NICU stay was associated with better neurocognitive outcomes. On the contrary, better weight gain beyond 12 months or 24 months of age was related to adverse cardiometabolic outcomes at school-age. As growth at different periods affects different outcomes in the later part of life, nutritional interventions and monitoring of growth might be established based on the age of the preterm infants.

## Supplementary Information

Supplementary Information

## Data Availability

The data that support the findings of the current study are available from the corresponding author on reasonable request.
