# Supplementary material for: Impact of size at birth and postnatal growth on metabolic and neurocognitive outcomes in prematurely born school-age children
Source: Sci Rep. 2021 Mar 25;11:6836. doi: 10.1038/s41598-021-86292-1 (PMC7994814; doi:10.1038/s41598-021-86292-1)
Supplement: Supplementary file 1 — Supplementary Information [file 41598_2021_86292_MOESM1_ESM.docx]

**Title**

**Impact of size at birth and postnatal growth on metabolic and neurocognitive outcomes in prematurely born school-age children**

**Authors**

Yoo Jinie Kim^1^, Seung Han Shin^1^, Eun Sun Lee^1^, Young Hwa Jung^1,2^, Young Ah Lee^1^, Choong Ho Shin^1^, Ee-Kyung Kim^1^, Han-Suk Kim^1^

| **<Supplementary table>**  **Supplementary Table S1. Feeding and activity** | | | |
| --- | --- | --- | --- |
|  | AGA (n=42) | SGA (n=14) | *p*-value |
| Any BM until (mo) | 5 (2-10) | 6 (2-15) | 0.280 |
| Complimentary food start (mo) | 8 (6-8) | 7 (6-8.5) | 0.726 |
| Any extra calorie add during BM or formula feeding | 4 (10.8) | 1 (7.1) | 1.000 |
| Intake at evaluation (calorie/day) | 1538.3 (1360.4-1757.2) | 1554.2 (1354.1-1723.8) | 0.872 |
| Moderate and vigorous activity minutes at evaluation (min/week) | 120 (60-390) | 120 (60-250) | 0.552 |
| Walk minutes (min/week) | 120 (30-270) | 150 (30-210) | 0.753 |
| Values are expressed as N (%) or Median (interquartile range) | | | |

Abbreviations: BM, breast milk; mo, month
